# Fractional Exhaled Nitric Oxide Falls Progressively During Treated Exacerbations of COPD and Is Discordant with Blood Eosinophil Counts

**DOI:** 10.3390/diagnostics16213567

**Published:** 2026-10-29

**Authors:** Dimitrios Potonos, Antonia Assioura, Athena Gogali, Emmanouil D. Oikonomou, Konstantinos Exarchos, Christos Kyriakopoulos, Konstantinos Kostikas

**Affiliations:** Respiratory Medicine Department, University of Ioannina, 45110 Ioannina, Greece; jimpotonos@yahoo.gr (D.P.); antonia.assioura@gmail.com (A.A.); athenagogali@yahoo.com (A.G.); oikonomou.emm@gmail.com (E.D.O.); kexarcho@gmail.com (K.E.); ckyriako123@gmail.com (C.K.)

**Keywords:** COPD, exacerbation, fractional exhaled nitric oxide, blood eosinophils, type-2 inflammation, treatable traits

## Abstract

**Background:** The behavior of fractional exhaled nitric oxide (FeNO) during treated COPD exacerbations and its agreement with blood eosinophil counts are poorly characterized. **Objectives:** To describe serial FeNO trajectories across hospitalized COPD exacerbations (AECOPDs), assess concordance between FeNO and blood eosinophils, and test whether these can predict length of stay (LOS). **Methods:** Prospective cohort of patients hospitalized for AECOPD. FeNO was measured on admission, on day 2 and at discharge; blood eosinophils and post-bronchodilator spirometry on admission and before discharge; and arterial blood gases on admission and on day 2. The admission measurements preceded the first dose of corticosteroid. All patients received guideline-directed treatment, including systemic corticosteroids. **Results:** Most patients were male (94.7%), with a mean age of 71.7 ± 8.3 years. FeNO fell from 16 (IQR 10–25) ppb on admission to 13.5 (7–20) ppb on day 2 and 9 (6–18) ppb at discharge (*p* < 0.001). Blood eosinophils fell from 60 (20–150) to 0 (0–40) cells/µL (*p* < 0.001), and FEV1 increased from 38% to 45% predicted (*p* < 0.001). Agreement between biomarkers on admission was poor (ρ = 0.21, *p* = 0.07; κ = 0.08, 95% CI −0.15 to +0.30), and 31 patients (40.8%) were classified differently by the two markers. Admission FeNO (ρ = 0.03, *p* = 0.79), ΔFeNO (ρ = −0.12, *p* = 0.31), and admission eosinophils (ρ = −0.08, *p* = 0.49) were not associated with LOS. Of the patients who were type-2-high on admission (FeNO ≥ 20 ppb), 57% had fallen below that threshold by day 2 and 62% by discharge (71% and 71%, respectively, at ≥25 ppb). Over the admission-to-discharge interval, the changes in the two markers were unrelated once each was adjusted for its own admission value (partial ρ = 0.05, *p* = 0.73), and there was no agreement on the direction of change (κ = 0.03). **Conclusions:** FeNO progressively declines during treated AECOPD, alongside a fall in the blood eosinophil count, so that the type-2 label assigned on admission is no longer valid for most patients 48 h later. FeNO and blood eosinophils identified largely non-overlapping patients, and no biomarker predicted length of stay.

## 1. Introduction

Exacerbations are the principal driver of morbidity, health-care utilization, and mortality in COPD [1]. Events severe enough to require hospitalization mark an inflection point in the natural history of the disease: they are followed by accelerated lung function decline, impaired health status, and a substantially increased risk of death, with the risk rising further as exacerbations recur [2,3]. Despite this prognostic weight, the in-hospital management of an exacerbation remains largely undifferentiated: systemic corticosteroids, bronchodilators and, where indicated, antibiotics are administered to nearly all patients irrespective of the inflammatory profile of the event. Identifying the subgroup with an eosinophilic, type-2 (T2)-driven exacerbation is therefore of practical interest, since these are the patients in whom corticosteroids are most likely to be effective and in whom their considerable adverse effects are most readily justified. Exacerbations are increasingly understood as heterogeneous events with distinct phenotypes, etiotypes and endotypes, and biomarker-defined subtypes are needed for individualised treatment [4].

Type 2 (T2) inflammation is now recognized as a treatable trait in COPD [5], and blood eosinophil count is a biomarker used in routine practice to guide inhaled corticosteroid therapy [6,7]. Fractional exhaled nitric oxide (FeNO) is an attractive complementary marker: it is non-invasive, provides immediate results at the bedside, and in asthma, reflects interleukin-13-driven epithelial inducible nitric oxide synthase activity [8]. Its behavior in COPD is much less well defined, and most available data come from stable states [9]. Sputum eosinophil counts remain the reference standard for airway eosinophilia and have been used to define eosinophilic exacerbation clusters, but induced sputum is impractical in an acutely breathless inpatient and is available in few centers [10]. This has driven the search for surrogate markers that can be obtained at the bedside within minutes of admission.

Several features of FeNO complicate its interpretation in COPD. Absolute values are lower than in asthma, current smoking suppresses nitric oxide output both acutely and chronically, and inhaled corticosteroids reduce it further, so the dynamic range in a treated COPD population is narrow [11,12]. Partitioned or multiple-flow measurements, which separate the alveolar nitric oxide concentration from bronchial nitric oxide flux, may capture peripheral airway inflammation that a single 50 mL/s value cannot [13], but they are not feasible at the bedside during an acute admission. These considerations make the timing and the context of a FeNO measurement at least as important as the value itself.

The evidence suggests that FeNO in COPD is not a fixed trait. In a large longitudinal cohort, intra-subject FeNO variability, rather than any single value, was associated with the risk and etiology of exacerbations [14]. Blood eosinophil counts behave similarly: repeated measurements in the same patients are frequently discordant across conventional thresholds, and more so in the stable state than during exacerbation [15,16]. Whether these two markers move together during a treated hospitalized COPD exacerbation, where systemic corticosteroids play a significant role, and whether they identify the same patients has been examined only rarely [17], and the question of whether single or multiple measurements are required in a given clinical setting remains open. A recent meta-analysis of 19 studies has confirmed at the group level that FeNO is elevated during an exacerbation and falls at clinical stability [18]; what happens to the classification of the individual patient over the first days of treatment, and how it relates to the blood eosinophil count measured at the same moments, has not been described.

In this preliminary study, we addressed three questions in a prospective cohort of patients hospitalized for an exacerbation of COPD (AECOPD): how FeNO behaves over the course of treatment, whether it identifies the same patients as the blood eosinophil count, and whether either marker or the early change in FeNO after treatment is started carries prognostic information about the length of hospital stay.

## 2. Materials and Methods

### 2.1. Study Design and Participants

We prospectively studied consecutive patients admitted with physician-diagnosed AECOPD to a single university respiratory department between July 2022 and March 2024. All patients included were 40 years or older, had previously diagnosed COPD with a post-bronchodilator FEV1/FVC ratio <0.70, were on optimal treatment by their treating physicians, were on their usual inhaled therapy, had received neither a systemic corticosteroid nor any additional inhaled corticosteroid for the specific exacerbation at the time of the admission measurements, and were able to perform spirometry and FeNO measurements on admission. Patients who required non-invasive or invasive ventilatory support at presentation were therefore not enrolled, since a reliable FeNO manoeuvre at a fixed expiratory flow cannot be performed during ventilatory support.

Patients with other significant respiratory conditions, including asthma, bronchiectasis, lung cancer, and radiographic pneumonia at the time of admission, were excluded. The study was approved by the Ethics Committee of the University Hospital of Ioannina (approval 18/23.09.2020), and all participants provided written informed consent. The department is a tertiary referral unit with a dedicated COPD service, and all patients were managed according to a standardized departmental protocol. An exacerbation was defined as an acute worsening of respiratory symptoms—increased dyspnea, sputum volume, or sputum purulence—beyond normal day-to-day variation and severe enough to require hospital admission [19]. The severity of airflow limitation was graded from the post-bronchodilator FEV1% predicted according to the GOLD spirometric classification [20]. Comorbidities were retrieved from the hospital record and from the patient’s regular medication list, and included arterial hypertension, diabetes mellitus, dyslipidemia, coronary heart disease, congestive heart failure, atrial fibrillation, chronic kidney disease, and gastro-esophageal reflux disease; the four most frequent are reported in Table 1.

### 2.2. Measurements

FeNO was measured at the bedside on admission, on the morning of day 2, and at discharge, using the NOBreath portable analyser (Bedfont Scientific Ltd., Kent, England, UK) at an expiratory flow of 50 mL/s, in accordance with ATS/ERS recommendations and the ERS technical standard for exhaled biomarkers [21,22]. Blood eosinophil counts and post-bronchodilator spirometry were obtained on admission and before discharge, and arterial blood gases on admission and on the morning of day 2. All patients received guideline-directed treatment including systemic corticosteroids (50 mg prednisolone equivalent), bronchodilators, and, where indicated, antibiotics [20]. Admission measurements were performed in the first two hours and preceded the first dose of systemic corticosteroids, which was given in the emergency department immediately afterwards. The day-2 measurement, taken on the following morning, was therefore obtained approximately 24 h after the start of systemic corticosteroid treatment. FeNO was measured by single-breath online analysis: after inhalation to near total lung capacity through a nitric oxide-scrubbing filter, patients exhaled at a constant flow of 50 mL/s against a fixed resistance, with visual feedback to maintain the target flow, and the plateau value was recorded in parts per billion (ppb). Because forced expiratory maneuvers and bronchodilators can transiently alter nitric oxide output, FeNO was always measured before spirometry and before the morning inhaled treatment.

Spirometry was performed at the bedside on admission and repeated before discharge. The forced expiratory volume in one second (FEV1) is the volume exhaled during the first second of a maximal forced expiration starting from full inspiration, and the forced vital capacity (FVC) is the total volume exhaled during that same maneuver; their ratio (FEV1/FVC) defines the presence of airflow obstruction, and a post-bronchodilator value below 0.70 was required for the diagnosis of COPD. FEV1 is expressed as a percentage of the predicted value, calculated from the Global Lung Function Initiative 2012 reference equations [23]; acceptability and repeatability criteria followed the ATS/ERS technical standard (Smart Pft USB Spirometer, Medical Equipment Europe) [24].

Symptom burden was assessed with the COPD Assessment Test (CAT), a validated eight-item questionnaire [25]. Each item is scored from 0 to 5, giving a total score between 0 and 40, with higher scores indicating a greater symptom burden. The CAT was completed on admission.

Arterial blood gases were sampled from the radial artery on admission, with the inspired oxygen fraction recorded at the time of sampling, and again on day 2. The following variables were recorded: arterial pH, the partial pressure of oxygen (PaO2, mmHg), the partial pressure of carbon dioxide (PaCO2, mmHg), the bicarbonate concentration, and lactate. Full blood counts were processed in the hospital’s central laboratory on an automated hematology analyzer, and the absolute blood eosinophil count in cells/µL was used throughout.

### 2.3. Statistical Analysis

Data are presented as median (interquartile range [IQR]) unless stated otherwise. Longitudinal changes were assessed using Friedman’s test, with Wilcoxon signed-rank post-hoc comparisons restricted to patients with all three measurements. Agreement between biomarkers was assessed with Spearman’s ρ and Cohen’s κ, using thresholds of FeNO ≥ 20 ppb and blood eosinophils ≥ 150 cells/µL to define type-2-high status [26]. No FeNO threshold has been validated specifically for COPD; these values are taken from the ATS clinical practice guideline [8] and have been applied in COPD populations, including for the prediction of exacerbation risk [27], and every analysis is therefore reported at both thresholds. ΔFeNO was defined as the day-2 value minus the admission value, such that negative values denoted a fall. Associations with length of stay (LOS) were assessed using Spearman’s ρ. In sensitivity analyses, ΔFeNO was categorized into tertiles and quartiles and dichotomized at several absolute and relative cutoffs. A two-sided *p* < 0.05 was considered significant. Descriptive statistics and the Friedman, Wilcoxon signed-rank, Mann–Whitney U and Spearman rank correlation analyses were performed using GraphPad Prism version 11 (GraphPad Software, Boston, MA, USA; www.graphpad.com). Wilson confidence intervals, the exact McNemar and exact binomial tests, the large-sample confidence interval for Cohen’s κ, partial Spearman correlations computed on rank residuals, and Oldham’s test for regression to the mean were computed in Python version 3.11 using the SciPy (version 1.17), statsmodels (version 0.15), NumPy (version 2.4) and pandas (version 3.0) libraries. No imputation was performed for missing data; every analysis used all patients with the relevant measurements available, and the corresponding n is reported alongside each result. Cohen’s κ is reported with its large-sample 95% confidence interval. To quantify the clinical consequence of the FeNO trajectory, patients classified as type-2-high on admission were re-classified on day 2 and at discharge using the same thresholds (≥20 and ≥25 ppb); the proportion re-classified as type-2-low is reported with 95% Wilson confidence intervals, and the change in the prevalence of type-2-high status between paired time points was tested with the exact McNemar test. Because a discharge FeNO measurement was not obtained in every patient, patients with and without that measurement were compared with respect to age, admission FeNO and length of stay using the Mann–Whitney U test, to establish whether the missingness was informative. A change in FeNO was considered clinically meaningful when it exceeded the criterion given in the ATS guideline—more than 10 ppb when the admission value was below 50 ppb or more than 20% when it was 50 ppb or above [8]—and the re-classification analysis was repeated, restricted to changes meeting that criterion. The relationship between the change in FeNO and the change in the blood eosinophil count, measured over the admission-to-discharge interval because that is the only interval over which both markers were obtained, was examined with Spearman’s ρ and, because each change score is mathematically coupled to its own baseline, also as a partial Spearman correlation computed on rank residuals after adjustment for both admission values; agreement on the direction of change was summarised with Cohen’s κ. Regression to the mean was assessed by Oldham’s method, in which the change is plotted against the mean of the two measurements rather than against the admission value, which removes the mathematical coupling between the two axes [28], and the symmetry of movement across each threshold was tested with the exact binomial test.

### 2.4. Use of Generative Artificial Intelligence

During the preparation of this work the authors used Claude Opus 5 (Anthropic) to assist with drafting and language editing of the manuscript, formatting to journal style, preparation of tables and figures from the analysis output, and consistency checks of all reported values against the source analysis output. The tool was not used to collect, generate or alter the primary clinical data, which were recorded prospectively by the investigators. After using this tool, the authors reviewed and edited the content as needed and take full responsibility for the content of the publication.

## 3. Results

### 3.1. Cohort Characteristics

Seventy-six patients were included in this study (Table 1). The cohort was predominantly male (72/76, 94.7%) and elderly (mean age 71.7, SD 8.3 years), heavily smoke-exposed (median 85 pack-years), with severe airflow obstruction (median FEV_1_ 39% predicted), and a high symptom burden (median CAT 21). All patients received inhaled therapy at the time of admission. The cohort carried a substantial burden of cardiovascular and metabolic comorbidity, with arterial hypertension in 44 patients (57.9%), diabetes mellitus in 23 (30.3%), coronary heart disease in 17 (22.4%), and congestive heart failure in 14 (18.4%). Admission arterial blood gases showed a median pH of 7.38 (7.34–7.42), a median PaCO2 of 37.8 mmHg (33.0–44.0), and a median PaO2 of 65.5 mmHg (58.0–75.5), indicating that most patients presented with hypoxemia in the absence of severe decompensated hypercapnia. The median length of stay was 5 days (4–7).

### 3.2. Serial FeNO, Eosinophils, and Lung Function

**Figure 1:**
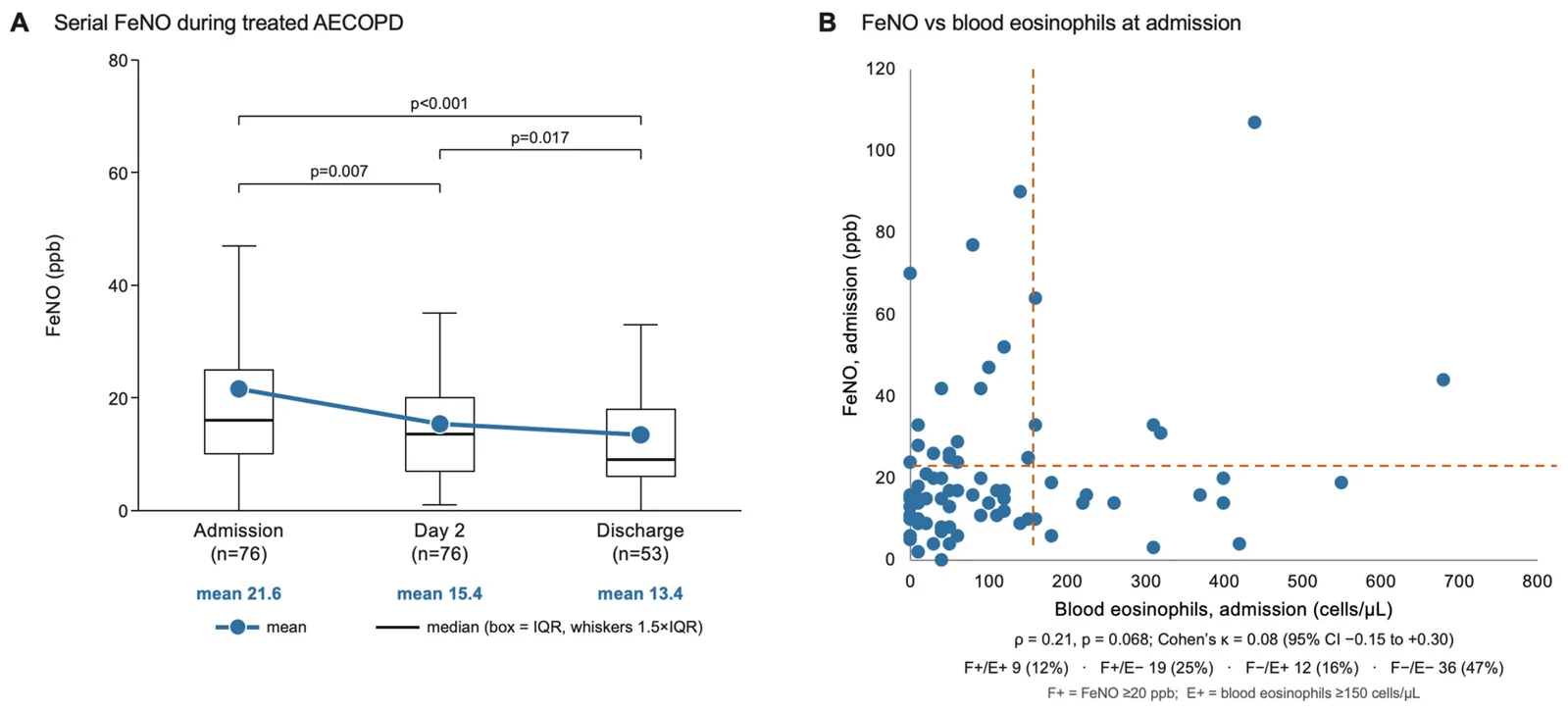
(**A**) FeNO levels on admission, day 2, and at discharge. Boxes show the median and IQR with 1.5 × IQR whiskers; the blue line connects the arithmetic mean. (**B**) Admission FeNO plotted against the admission blood eosinophil count; dashed lines mark the FeNO ≥ 20 ppb and eosinophil count ≥ 150 cells/µL thresholds.

FeNO progressively decreased during admission: 16 ppb (10–25) on admission, 13.5 ppb (7–20) on day 2, and 9 ppb (6–18) at discharge (*p* < 0.001 among the 53 patients with all three measurements; pairwise Wilcoxon signed-rank tests restricted to the same 53 complete cases and Bonferroni-corrected for three comparisons: admission vs. day 2 *p* = 0.007, day 2 vs. discharge *p* = 0.017, admission vs. discharge *p* < 0.001; Figure 1A). The median relative fall from admission to discharge was 33% (0–64), indicating that a substantial minority showed no decrease. Over the same interval, the blood eosinophil count fell from 60 (20–150) to 0 (0–40) cells/µL (n = 71, *p* < 0.001), with 36 of 71 patients (51%) having an undetectable count at discharge, and FEV1 rose from 38% to 45% predicted in the 58 patients with both measurements (*p* < 0.001; the median admission FEV1 among all 69 patients in whom it was obtained was 39% predicted, which is the figure reported in Table 1). The trajectory is shown in Figure 1A, in which the boxes summarize the distribution at each time point and the blue line follows the arithmetic mean. The decline is evident in both summary measures—medians of 16, 13.5, and 9 ppb, and means of 21.6, 15.4, and 13.4 ppb—and the upper quartile contracted from 25 ppb on admission to 18 ppb at discharge, indicating that the fall was not confined to the patients with the highest admission values. A discharge FeNO measurement was available in 53 of the 76 patients (69.7%). Those in whom it was obtained did not differ from the remainder in age (*p* = 0.24) or admission FeNO (*p* = 0.44), but had a longer hospital stay (median 6 vs. 4 days, *p* = 0.004); the three-point trajectory therefore describes the patients whose admission lasted long enough for a third measurement to be made and should not be extrapolated to the shortest admissions.

### 3.3. Agreement Between FeNO and Blood Eosinophils

The agreement between the two type-2 biomarkers at admission was poor (Figure 1B). Twenty-eight patients (36.8%) had an FeNO ≥ 20 ppb and 21 (27.6%) had an eosinophil count ≥ 150 cells/µL; however, the two measures were only weakly correlated (ρ = 0.21, *p* = 0.07) and classified patients no better than chance (Cohen’s κ = 0.08, 95% CI −0.15 to +0.30). Only nine patients (11.8%) were type-2-high by both criteria; 19 (25.0%) were FeNO-high but eosinophil-low, 12 (15.8%) were the reverse, and 36 (47.4%) were low by both. Figure 1B plots each patient as a single point in the plane defined by the two admission biomarkers, with the dashed lines marking the FeNO ≥ 20 ppb and eosinophil ≥ 150 cells/µL thresholds and dividing the plane into four quadrants. The upper-right quadrant, containing the patients who are type-2-high by both criteria, is the most sparsely populated: those nine patients are outnumbered more than twofold by the 19 who are FeNO-high but eosinophil-low in the upper-left quadrant, while the largest single group lies in the lower-left quadrant and is low by both criteria. Taken together, 31 of the 76 patients (40.8%) were assigned a different type-2 label by the two markers. Whether the two markers also moved together within the individual patient is examined in Section 3.5.

### 3.4. Reclassification of Type-2 Status During Treatment

**Figure 2:**
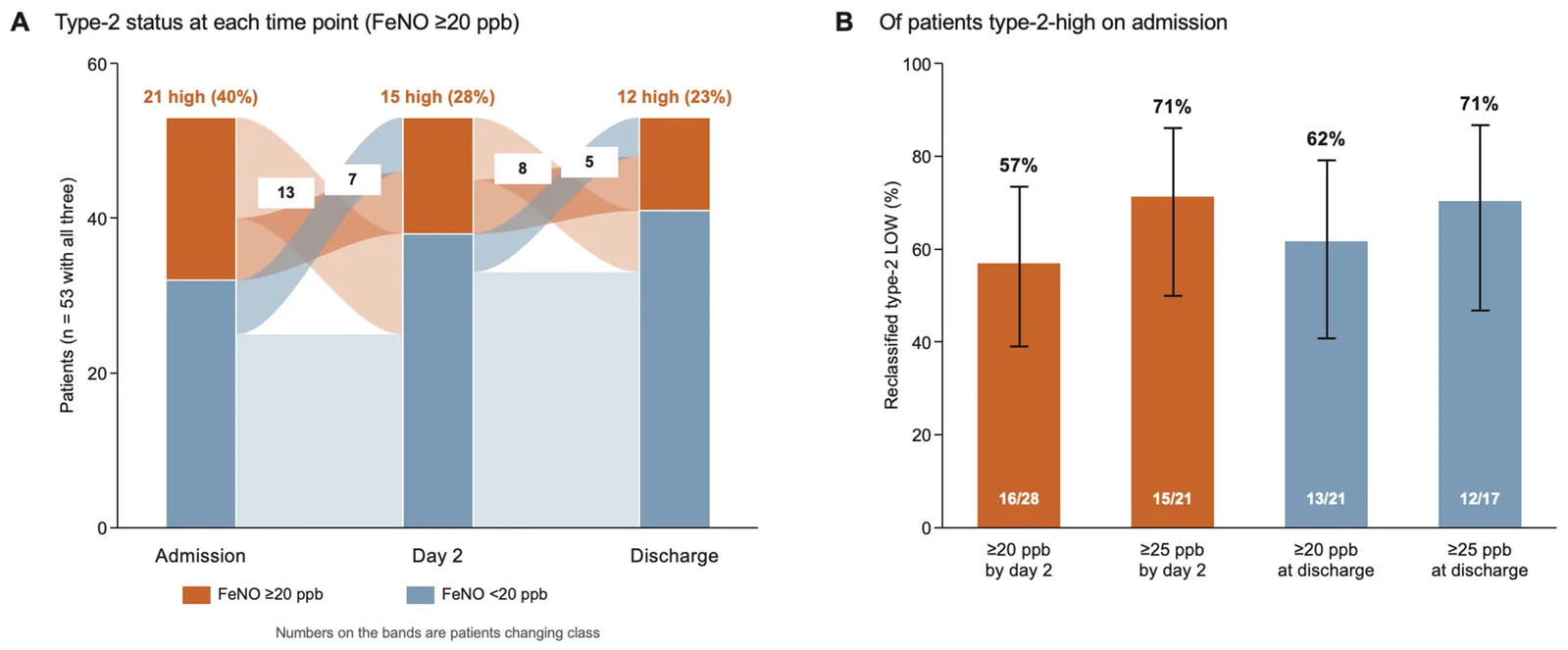
(**A**) Type-2 status (FeNO ≥ 20 ppb) at admission, on day 2 and at discharge in the 53 patients with all three measurements; the bands show individual transitions and the numbers indicate the patients changing class. (**B**) Proportion of the patients who were type-2-high on admission and were re-classified as type-2-low on day 2 and at discharge, at both thresholds, with 95% Wilson confidence intervals. The denominators differ between the day-2 and the discharge bars because the discharge measurement was not available in every patient.

Because FeNO fell in most patients, the type-2 label assigned on admission was frequently not the label that the same patient would have received one or two days later (Figure 2). Of the 28 patients who were type-2-high on admission at the ≥20 ppb threshold, 16 (57.1%, 95% CI 39.1–73.5) had fallen below that threshold by day 2, and 13 of the 21 with a discharge measurement (61.9%, 95% CI 40.9–79.2) were below it at discharge. At the more specific ≥ 25 ppb threshold the movement was larger: 15 of 21 patients (71.4%, 95% CI 50.0–86.2) were re-classified by day 2 and 12 of 17 (70.6%, 95% CI 46.9–86.7) at discharge (Figure 2B). The prevalence of type-2-high status consequently fell from 36.8% on admission to 26.3% on day 2 and 22.6% at discharge at the ≥20 ppb threshold (exact McNemar *p* = 0.15 and *p* = 0.049, respectively) and from 27.6% to 13.2% and 11.3% at the ≥25 ppb threshold (*p* = 0.019 and *p* = 0.003).

Figure 2A displays these movements at the level of the individual patient in the 53 patients with all three measurements. Twenty-one patients (40%) were type-2-high on admission, 15 (28%) on day 2 and 12 (23%) at discharge; however, the change in these marginal totals conceals substantially more individual movement than the totals imply, since 13 patients crossed from high to low between admission and day 2 while 7 crossed in the opposite direction, and a further 8 crossed from high to low and 5 from low to high between day 2 and discharge. Agreement between the admission and the day-2 classification was correspondingly modest: of the 36 patients who were type-2-high at either of the two time points, only 12 (33.3%) were type-2-high at both.

The same instability applied to a composite definition. Forty of the 76 patients (52.6%) were type-2-high on admission when either criterion was allowed to qualify (FeNO ≥ 20 ppb or blood eosinophils ≥ 150 cells/µL), and 12 of those 40 (30.0%) would no longer have qualified had the day-2 rather than the admission FeNO been used.

### 3.5. Clinically Meaningful Change, Regression to the Mean, and the Relationship Between the Two Biomarkers

**Figure 3:**
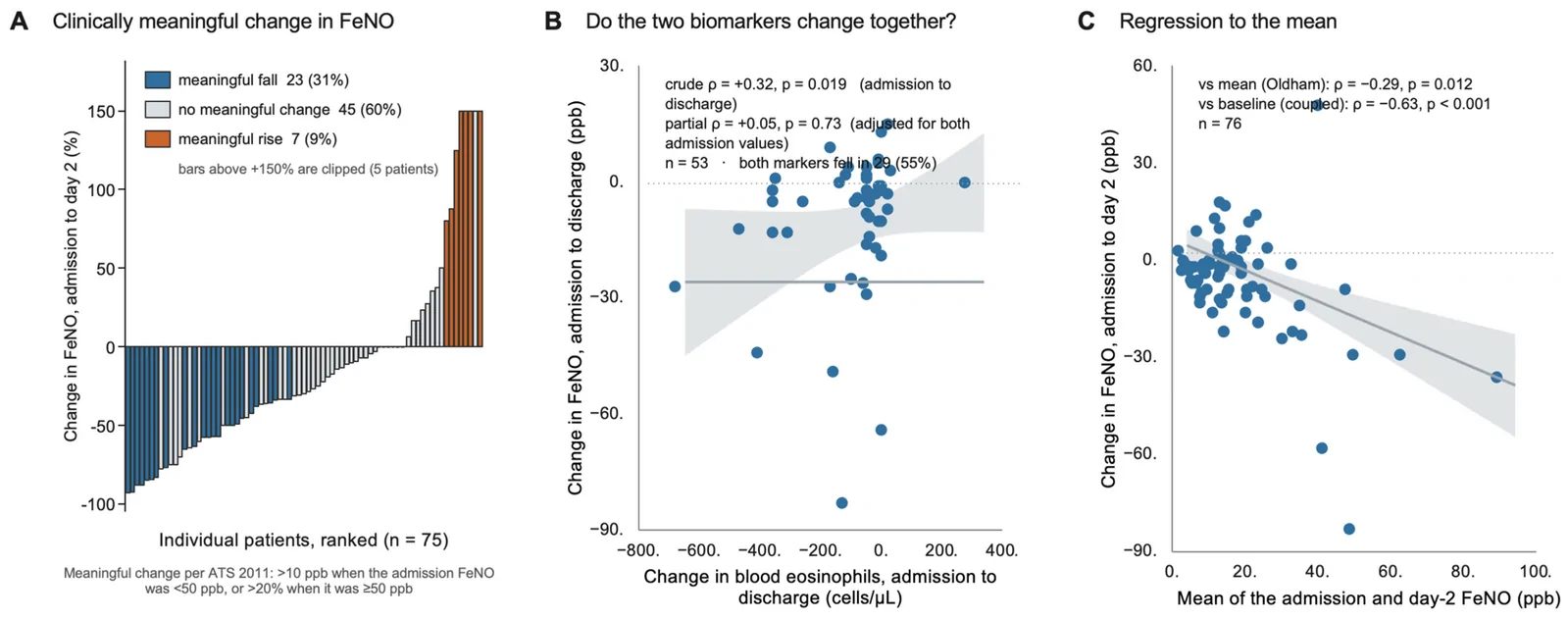
(**A**) Change in FeNO between admission and day 2 in each patient, ranked, with the bars colored according to whether the change met the ATS criterion for a clinically meaningful difference (more than 10 ppb when the admission value was below 50 ppb, or more than 20% when it was 50 ppb or above). (**B**) Change in the blood eosinophil count plotted against the change in FeNO, both measured from admission to discharge, which is the only interval over which both markers were obtained, showing the crude correlation and the correlation adjusted for both admission values; panels A and C describe the change in FeNO between admission and day 2. (**C**) Oldham plot: the change in FeNO plotted against the mean of the admission and day-2 values, which removes the mathematical coupling that is present when the change is plotted against the admission value. Grey lines are least-squares fits with their 95% confidence bands.

Applying the criterion given in the ATS guideline for a clinically meaningful change in FeNO, 23 of the 76 patients (30.3%) had a meaningful fall between admission and day 2, 7 (9.2%) had a meaningful rise and 46 (60.5%) had no meaningful change; between admission and discharge the corresponding figures were 19 of 53 (35.8%), 2 (3.8%) and 32 (60.4%) (Figure 3A). Restricting the re-classification analysis to changes meeting this criterion did not materially alter it. Of the 16 patients re-classified from type-2-high to type-2-low by day 2 at the ≥20 ppb threshold, the fall exceeded the ATS criterion in 11 (69%); of the 13 re-classified at discharge, in 11 (85%); of the 15 re-classified by day 2 at the ≥25 ppb threshold, in 12 (80%); and of the 12 re-classified at discharge at that threshold, in all 12 (100%).

Because the change in FeNO is mathematically coupled to the admission value, regression to the mean was assessed by Oldham’s method. Plotted against the mean of the admission and day-2 values, the change remained inversely associated (ρ = −0.29, *p* = 0.012; Figure 3C), so a contribution from regression to the mean cannot be excluded; the coupled statistic, the change plotted against the admission value, was ρ = −0.63 and is reported only for comparison. However, movement across the thresholds was asymmetric at the more specific cut-point: among the 53 patients with all three measurements, 12 crossed downward and 2 upward across ≥25 ppb between admission and day 2 (exact binomial *p* = 0.013), whereas across ≥20 ppb the movement was 13 down and 7 up (*p* = 0.26). The median within-patient coefficient of variation in FeNO across the three time points was 0.46.

The changes in the two biomarkers were not related within the individual patient (Figure 3B). Blood eosinophils were measured on admission and at discharge, so this comparison was made over that interval, which is the only one for which both markers are available in the same patients. In the 53 patients with both a discharge FeNO and a discharge eosinophil count, the crude correlation between the two changes was ρ = +0.32 (*p* = 0.019); however, each change was strongly coupled to its own admission value (ρ = −0.70 for FeNO and ρ = −0.82 for eosinophils), and after adjustment for both admission values the association disappeared (ρ = +0.05, 95% CI −0.22 to +0.32, *p* = 0.73). Both markers fell in 29 of the 53 patients (54.7%), which is what the marginal frequencies would predict by chance, and agreement on the direction of change was negligible (κ = 0.03). Agreement between the two markers measured cross-sectionally at discharge could not be assessed meaningfully, since only 2 of the 71 patients (2.8%) still had an eosinophil count ≥ 150 cells/µL after treatment. Both markers therefore fell at the level of the group while moving independently within patients.

### 3.6. Biomarkers and Length of Stay

**Figure 4:**
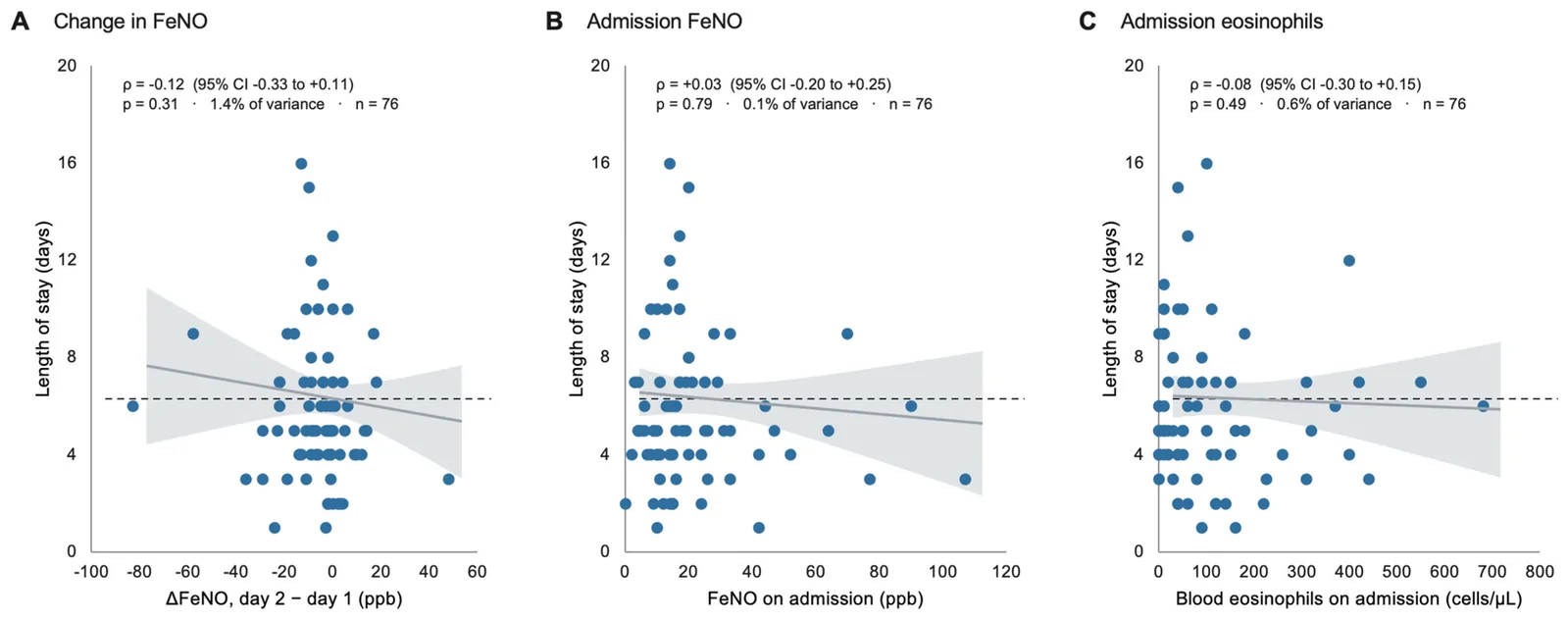
Length of hospital stay plotted against (**A**) ΔFeNO (day 2–day 1), (**B**) FeNO on admission, and (**C**) blood eosinophil count on admission. The grey lines are least-squares fits with their 95% confidence bands; the dashed line marks the mean length of stay, which lies within the confidence band across the whole range of every biomarker.

None of the biomarkers were associated with length of stay (median 5 days, 4–7; Figure 4): admission FeNO ρ = 0.03, *p* = 0.79; ΔFeNO ρ = −0.12, *p* = 0.31; admission blood eosinophils ρ = −0.08, *p* = 0.49. The results for ΔFeNO remained unchanged when dichotomized at different cut-offs. In an exploratory analysis, a higher admission eosinophil count was associated with a smaller improvement in FEV_1_ (ρ = −0.29, *p* = 0.026, *n* = 58). These relationships are displayed in Figure 4. In each panel—Figure 4A for ΔFeNO, Figure 4B for admission FeNO, and Figure 4C for the admission blood eosinophil count—the fitted least-squares line is almost flat, and its 95% confidence band encloses the horizontal dashed line marking the mean length of stay across the entire observed range of the biomarker. This is the graphical counterpart of the absence of a statistical association: the data are compatible with no relationship at any value of any of the three markers, and each marker accounted for less than 2% of the variance in length of stay.

## 4. Discussion

In this preliminary analysis, we showed that FeNO levels declined progressively during treated AECOPD, alongside a fall in the blood eosinophil count, and that this decline was large enough to change the type-2 label of most patients who were type-2-high on admission within 48 h. FeNO and blood eosinophils identified largely non-overlapping patients, and no biomarker predicted length of stay. Given the growing interest in FeNO in COPD management [29], this study attempted to highlight the potential role of FeNO and blood eosinophil counts during COPD exacerbations.

Three observations from these results are of potential clinical importance. First, FeNO is not a static property of the exacerbating patient; it falls by roughly a third within a few days of treatment, so any single value depends heavily on when it was measured relative to the start of therapy. This extends to the inpatient setting the observation that it is the variability of FeNO, rather than any single reading, that carries information in COPD [14], and it implies that studies phenotyping patients on an admission FeNO must state that timing explicitly. Second, FeNO and blood eosinophil counts identified largely different individuals. Agreement here was not clinically relevant (κ = 0.08, 95% CI −0.15 to +0.30), and three-quarters of patients with high FeNO levels were not eosinophilic. It is worth being explicit about what this confidence interval does and does not mean, because correlation and agreement are distinct quantities [30]. The width of the interval is a function of the sample size, whereas its location is a property of the agreement itself; a larger cohort would narrow the interval around κ ≈ 0.08 but would not move it toward the range (κ > 0.40) at which two tests may reasonably be treated as interchangeable [31]. The finding of interest here is not the *p* value attached to ρ but the fact that 40.8% of patients were labelled differently by the two markers, a proportion that is estimated with useful precision in a cohort of this size. This mirrors population-level data showing only partial overlap between the two markers in chronic airway disease [32,33] and the finding in severe asthma that combining the two adds little to either marker alone [34]. This is important as type-2-directed therapies enter COPD practice, where eligibility is currently defined by eosinophil count alone [35,36,37]. Neither marker should be treated as a stable label, as eosinophil counts are discordant on repeat measurements in stable and exacerbating COPD [15,16,38]; therefore, the discordance we observed is between two moving quantities rather than between a candidate marker and a fixed standard. Third, none of the three measures—admission FeNO, ΔFeNO, or blood eosinophil count—could predict the length of hospital stay.

Our eosinophil results deserve additional comment because a larger prospective study of 388 patients hospitalized for AECOPD found that a higher admission eosinophil count was associated with less dyspnea, lower CRP, and shorter stay [39]. The direction of our estimate is the same (ρ = −0.08), but with 76 patients, we likely did not have enough power to detect an effect of that size, and our finding should be read as consistent with, rather than contradicting, our previous larger study. More broadly, exhaled biomarkers in AECOPD remain insufficiently validated for clinical use. Fourth, and in our view the observation with the clearest practical consequence, the type-2 label itself proved unstable: of the patients who met the FeNO ≥ 20 ppb criterion on admission, 57% no longer met it on day 2 and 62% no longer met it at discharge, and the figures at the ≥25 ppb threshold were higher still. A patient’s type-2 status during a hospitalized exacerbation is therefore not a property of the patient but a property of the patient and the hour at which the sample was taken.

Our findings can be placed against a recent meta-analysis of 19 studies of FeNO in AECOPD [18]. In the 14 studies that measured patients during the acute phase and again after clinical stabilization, FeNO fell by a pooled mean difference of 6.27 ppb (95% CI 3.57–8.97), and the fall was larger in patients with more severe airflow obstruction. The decline we observed is of almost exactly that magnitude—a mean of 21.6 ppb on admission against 13.4 ppb at discharge, a difference of 8.2 ppb. The comparator in the pooled studies was clinical stability, usually reached weeks or months after the event, and the authors report that the interval between the two determinations did not influence the effect size; our data refine that observation by showing that most of the fall has already occurred by the morning of day 2, within roughly 24 h of the first dose of systemic corticosteroid.

Río Ramírez and colleagues in their study addressed the question in a design close to ours [17]. In 50 patients admitted with AECOPD, FeNO was measured on arrival in the emergency department, at discharge and in stability three to six months later, and a correlation of r = 0.70 was reported between FeNO and the blood eosinophil count on arrival, considerably stronger than the agreement we observed. The two results are comparable, yet with some differences. Their cohort comprised 26% asthma–COPD overlap, and those patients had markedly higher FeNO (73 ppb) and eosinophil counts (508 cells/mm^3^) than those with chronic bronchitis or emphysema (23–27 ppb and 159–184 cells/mm^3^). Patients with asthma were excluded from our cohort, whose median admission FeNO was 16 ppb, so the question we answer is clinically relevant to patients with COPD admitted with an exacerbation. Moreover, both studies found no association between either marker and the length of hospital stay. What our study adds is an early post-treatment measurement, which allows the speed of re-classification to be quantified, and identify early response to treatment.

A plausible explanation for the discordance between the two markers is that they report on different compartments and are governed by different processes. FeNO reflects inducible nitric oxide synthase activity in the airway epithelium, driven largely by interleukin-13, and is therefore a proximate readout of type-2 signaling in the airway wall. The blood eosinophil count reflects the circulating pool, which is determined by bone marrow output, margination, and tissue recruitment and is exquisitely sensitive to both endogenous and exogenous corticosteroids. A patient may therefore be epithelially type-2 active while the circulating count is low, or the converse. Smoking introduces a further dissociation, since current smoking suppresses nitric oxide output in COPD without a comparable effect on the eosinophil count [11]. Smoking status at the time of admission was not recorded in our database, and the direction of the resulting bias deserves comment. Current smoking suppresses exhaled nitric oxide; therefore, any patient who was smoking at presentation would have had a lower admission value, which compresses the dynamic range of the marker and biases both the agreement analysis and the association with length of stay toward the null. Patients necessarily abstained from smoking in hospital, and since the acute suppressive effect resolves within hours, abstinence will have exerted an upward pressure on FeNO over the course of the admission. Both effects oppose the fall we observed, so the decline we report, and the proportion of patients re-classified, are rather conservative estimates; the same mechanism offers a plausible partial explanation for the seven patients in whom FeNO rose by more than the ATS criterion.

The simultaneous decline in FeNO and eosinophils is most plausibly attributable to systemic corticosteroids, which are well known to suppress both [40], compounded by resolution of the exacerbation itself; our design cannot separate these contributions. The inverse association between admission eosinophil levels and FEV_1_ recovery runs contrary to trial evidence that eosinophilic exacerbations respond better to corticosteroids [41]. Given the sample size, the absence of adjustment for baseline lung function, and the potential for regression to the mean, we regard it as hypothesis-generating only. Our observational design cannot separate the effect of corticosteroid treatment from the natural resolution of the exacerbation, since an untreated comparison group would not be ethical in this population; and although both markers fell at the level of the group, their movements within individual patients were unrelated, so the two declines should not be described as a single process.

A reasonable consideration is related to how much of the movement we describe reflects treatment and how much reflects the inherent variability of the measurement. The re-classification was not a matter of values drifting across a threshold: among the patients who moved from T2-high to T2-low, the change also exceeded the ATS criterion for a clinically meaningful difference in 69% at the ≥20 ppb threshold by day 2 and in all of those reclassified at the ≥25 ppb threshold at discharge. Movement across the ≥25 ppb threshold was asymmetric, with 12 patients crossing downward and 2 upward between admission and day 2 (*p* = 0.013), which regression to the mean alone would not produce, although across the ≥20 ppb threshold it was not (13 down, 7 up, *p* = 0.26). Against this, Oldham’s test showed that the change remained associated with the mean of the two measurements (ρ = −0.29, *p* = 0.012). Therefore, a contribution from regression to the mean cannot be excluded. The within-patient coefficient of variation in FeNO across the three time points was 0.46, which is of the same order as the short-term variability reported in stable COPD, where more than 30% of patients crossed the 25 ppb threshold at least once over an 18-day period of measurement [42] and where within-day variability alone has been judged sufficient to question the use of a single reading [43]. We therefore cannot claim that the reduction is entirely due to treatment, but in part may be attributed to measurement variability, that is a further argument against acting on a single FeNO value.

Several practical implications follow. First, if FeNO is to be used to phenotype an exacerbating patient, the measurement must be obtained before the first dose of systemic corticosteroid and its timing reported explicitly; a value obtained 48 h into treatment describes the response to therapy rather than the phenotype of the event. In a ward in which the first dose of corticosteroid is usually given in the emergency department, this is an operational requirement rather than a methodological nicety, and it is one that most retrospective datasets cannot satisfy. Second, because the two markers disagree in approximately half of the patients, a composite definition of type-2 status will identify a materially different population depending on whether “either” or “both” criteria are required, and this choice should be pre-specified rather than made post hoc; in our cohort 40 patients (52.6%) met an “either” definition on admission, and 12 of those 40 (30.0%) would have been excluded had the day-2 rather than the admission FeNO been used. Third, for trials that enrich for type-2 inflammation at the time of an exacerbation, the eligibility window matters as much as the threshold: a protocol that permits screening at any point during the first 48 h of admission will, on our data, enroll a materially different population from one that requires screening before the first dose of corticosteroid, and the resulting misclassification is non-random because it falls disproportionately on the patients who respond to treatment. Fourth, and conversely, the instability we describe is a reason to measure FeNO more than once rather than a reason to abandon it: the admission value and the day-2 value answer different clinical questions, the first describing the untreated event and the second the early response to therapy. Whether the FeNO value at discharge, which reflects residual airway inflammation after treatment, carries information about subsequent exacerbation risk is an obvious next question, and one that our design, which lacked post-discharge follow-up, cannot address. The principal strength of the present analysis is that the admission measurement preceded the first dose of systemic corticosteroid, so the baseline value reflects the untreated exacerbation rather than an early response to treatment; few studies of FeNO in hospitalized AECOPD have been able to guarantee this. This may be important for clinical practice. Eligibility for T2-directed biologic treatments in COPD is currently defined by blood eosinophil counts alone [44], a convention that has been questioned [45], and whether FeNO adds anything to the eosinophil count for predicting the response to a type-2 biologic is now being examined directly [46]. Where that assessment is made around a hospitalization [47], our data indicate that the day on which the sample is taken will change which patients qualify. Moreover, serial rather than single FeNO measurements carry information that one reading does not, both during an admission and in the stable state [48].

This preliminary study had some limitations. It is a single-center cohort study conducted in a tertiary hospital with a special interest in COPD. The cohort was 94.7% male, which is close to our clinical experience but limits generalizability to women. All patients were receiving inhaled corticosteroids as regular treatment prior to their admission, which suppress FeNO and compress its dynamic range [12]; however, none had received systemic or inhaled corticosteroids at the time of the first measurements of FeNO or blood eosinophils on admission, which represents an important strength of our study. The recent smoking status at the time of measurement could not be recorded, and acute smoking lowers FeNO [11]. Discharge FeNO was missing in 23 of 76 patients for feasibility reasons. Importantly, with 76 patients, the study had 80% power to detect a correlation of ρ ≥ 0.32 with LOS; therefore, a weak true association cannot be excluded. Finally, we cannot exclude that length of stay may be influenced by administrative and social factors; however, we made every possible effort to use similar criteria in all patients, as this is a single-department study with standardized practice. Two further limitations bear on the interpretation of the agreement analysis. A discharge FeNO was available in 69.7% of patients, and those patients had longer admissions, so the trajectory and re-classification analyses are conditioned on a stay long enough to permit a third measurement. In addition, a cohort of this size estimates κ imprecisely: approximately 380 patients would be required to estimate κ to within ±0.10, and approximately 176 to detect a correlation of ρ = 0.21 between the two markers at 80% power. We would emphasize, however, that such a study would establish the precision of the estimate of agreement rather than improve the agreement itself, and the point estimate obtained here is already well below the range that would justify using the two markers interchangeably.

Three further limitations should be stated. First, we did not measure sputum eosinophils, which remain the reference standard for airway eosinophilia; in their absence this study demonstrates disagreement between two surrogate markers rather than the diagnostic accuracy of either, and no statement about the sensitivity or specificity of FeNO or of the blood eosinophil count can be made from these data. Second, the thresholds of 20 and 25 ppb are not validated for COPD, which is why every analysis is reported at both; the conclusions are the same at each. Third, patients requiring ventilatory support were not enrolled, and because the need for ventilatory support is the principal determinant of a prolonged admission, the range of the length of stay in our cohort was correspondingly narrow—a median of 5 days with an interquartile width of 3 days, 80.3% of patients discharged within a week and only 6.6% staying beyond 10 days. The power to detect an association between an admission biomarker and so restricted an outcome is low whatever the biomarker, and our null result should therefore be read as applying to patients whose exacerbation is severe enough to require admission but not severe enough to require ventilatory support, rather than to AECOPD in general. This also reconciles our result with the larger unselected cohort cited above and with pooled evidence that the admission eosinophil count relates to the outcome of an exacerbation [49]; an adequately powered test of the prognostic question requires a cohort that includes patients receiving non-invasive ventilation.

## 5. Conclusions

FeNO declines progressively during a treated exacerbation of COPD, alongside a fall in the blood eosinophil count and recovery of lung function and identifies a largely different group of patients from that identified by blood eosinophil counts. The magnitude of that decline is sufficient to re-classify the majority of patients who are type-2-high on admission within 48 h, so the timing of measurement is critical for the interpretation of FeNO in this setting, and the two type-2 biomarkers should not be used interchangeably. Neither the admission values nor the early changes in FeNO predicted the length of hospital stay. Serial rather than single measurements, obtained at defined time points relative to the start of corticosteroid therapy, are likely to be required if FeNO is to contribute to the phenotyping of exacerbations, and larger cohorts with post-discharge follow-up will be needed to establish whether either marker carries prognostic information beyond the index admission.

## Figures and Tables

**Table 1:** Baseline characteristics, biomarkers, and lung function of 76 patients hospitalized for exacerbation of COPD.

Characteristic	Value	n
Age, years—mean (SD)	71.7 (8.3)	76
Male sex—n (%)	72 (94.7)	76
Body mass index, kg/m^2^	26.7 (22.3–29.8)	74
Smoking history, pack-years	85.0 (50.0–100.0)	74
CAT score	21.0 (15.2–26.0)	74
Arterial pH	7.38 (7.34–7.42)	76
PaCO_2_, mmHg	37.8 (33.0–44.0)	76
PaO_2_, mmHg	65.5 (58.0–75.5)	76
Self-reported allergy—n (%)	8 (10.5)	76
Arterial hypertension—n (%)	44 (57.9)	76
Diabetes mellitus—n (%)	23 (30.3)	76
Coronary heart disease—n (%)	17 (22.4)	76
Congestive heart failure—n (%)	14 (18.4)	76
Length of stay, days	5.0 (4.0–7.0)	76
**Biomarkers**		
FeNO, admission (ppb)	16.0 (10.0–25.0)	76
FeNO, day 2 (ppb)	13.5 (7.0–20.0)	76
FeNO, discharge (ppb)	9.0 (6.0–18.0)	53
ΔFeNO, day 2—day 1 (ppb)	−3.0 (−11.0 to +0.0)	76
FeNO ≥ 20 ppb at admission—n (%)	28 (36.8)	76
FeNO ≥ 25 ppb at admission—n (%)	21 (27.6)	76
Blood eosinophils, admission (cells/µL)	60.0 (20.0–150.0)	76
Blood eosinophils, discharge (cells/µL)	0.0 (0.0–40.0)	71
Eosinophils ≥ 150 cells/µL at admission—n (%)	21 (27.6)	76
Eosinophils ≥ 300 cells/µL at admission—n (%)	10 (13.2)	76
**Lung function**		
FEV_1_, % predicted (admission)	39.0 (29.0–49.0)	69

Data are presented as median (IQR) unless otherwise stated. The n column indicates the number of patients with available data for that variable. CAT: COPD Assessment Test; FeNO: fractional exhaled nitric oxide.

## Data Availability

The de-identified data supporting the reported results are available from the corresponding author on reasonable request.
